# MicroRNA‐188 inhibits biological activity of lung cancer stem cells through targeting *MDK* and mediating the Hippo pathway

**DOI:** 10.1113/EP088704

**Published:** 2020-07-08

**Authors:** Xiaolin Yang, Baogang Wang, Wenbo Chen, Xiaxia Man

**Affiliations:** ^1^ Department of Geriatrics The First Hospital of Jilin University Changchun Jilin 130021 PR China; ^2^ Department of Cardiac Surgery The First Hospital of Jilin University Changchun Jilin 130021 PR China; ^3^ Department of Emergency The First Hospital of Jilin University Changchun Jilin 130021 PR China; ^4^ Department of Oncological Gynecology The First Hospital of Jilin University Changchun Jilin 130021 PR China

**Keywords:** biological activity, Hippo pathway, lung cancer, *MDK*, microRNA‐188, tumour stem cells

## Abstract

**New Findings:**

- **What is the central question of this study?**
  The aim was to investigate the function of microRNA‐188 in the biological characteristics of lung cancer stem cells and the molecular mechanisms involved.
- **What is the main finding and its importance?**
  This study highlights a new molecular mechanism involving microRNA‐188, *MDK* and the Hippo signalling pathway that plays a suppressive role in biological activity of lung cancer stem cells. This finding might offer new insights into gene‐based therapy for lung cancer.

**Abstract:**

MicroRNAs (miRNAs) have been implicated in lung cancer and reported as new promising diagnostic and therapeutic tools for cancer control. Here, we investigated the action of microRNA‐188 (*miR‐188*) in lung cancer stem cells. We first tested *miR‐188* expression in clinical samples of lung cancer patients, and a low expression profile of *miR‐188* was found. Next, we analysed the role of *miR‐188* in lung cancer stem cells with cell growth assays. To verify the *in vitro* results, we used a xenograft model to validate the capability of *miR‐188* in tumorigenesis. Overexpression of *miR‐188* reduced viability and metastasis of cancer stem cells. Similar results were reproduced *in vivo*, where overexpression of *miR‐188* retarded tumour growth in mice. We also identified *MDK* as a target of *miR‐188*, and overexpression of *MDK* was found in lung cancer samples. Overexpressed *MDK* promoted the malignant behaviours of lung cancer stem cells. In addition, the Hippo pathway was found to be inactivated in lung cancer tissues, presenting as increased levels of YAP and TAZ. Suppression of the Hippo pathway also enhanced lung cancer stem cell activity and promoted the growth of xenograft tumours. To sum up, our results reveal that *miR‐188* inhibits the malignant behaviours of lung cancer stem cells and the growth of xenograft tumours. This study might offer new insights into gene‐based therapies for cancer.

## Graphical abstract

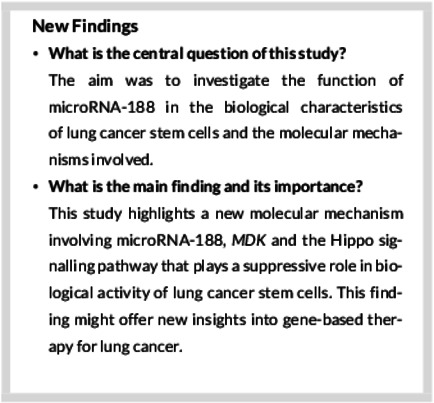

## 1 INTRODUCTION

Lung cancer is one of the most prevalent tumours around the world and a main contributor to cancer‐related death, among which non‐small cell lung cancer (NSCLC) is the main type of lung cancer, accounting for 80–85% of all cases (Qiang et al., 2020). The pathogenesis of lung cancer is multifactorial, including both environmental and genetic factors, and the occurrence of lung cancer is associated with the modulation of tumour suppressor genes and oncogenes (Yu et al., 2020). In view of the insidious onset of lung cancer, most patients are in an advanced stage at the time of first diagnosis, rendering treatment difficult (Zhang, Yuan, Gao, & Zhang, 2018). More importantly, patients with such malignancies usually have a poor prognosis, and the 5‐year survival rate is very low owing to the lack of timely treatment and limited access to early detection (Xiaoguang et al., 2017). Thus, there is an urgent need to identify and develop new therapeutic options to control lung cancer.

Most microRNAs (miRNAs) bind to complementary sites in the target mRNAs in 3′ untranslated regions (UTRs) to inhibit gene expression via either repression of protein translation or directing mRNA degradation (Pillai, 2005). In cancer research, microRNA‐188 (*miR‐188*) has been illustrated as tumour inhibitor in different cancer models, including prostate cancer, oral squamous cell carcinoma and hepatocellular carcinoma (Fang et al., 2015; Wang & Liu, 2016; Zhang et al., 2015). Also, it is reported that *miR‐188* is downregulated in lung cancer cells (Zhao et al., 2018). Midkine (*MDK*) is known as a heparin‐binding growth factor that is upregulated in some malignant tumours, including lung cancers (Hao et al., 2013). The human *MDK* gene is positioned on chromosome 11p11.2, and there are four exons in the coding frames of the protein (Muramatsu, 2002). Interestingly, *MDK* is of significance in human tumour processes and in biological processes such as enhancement of fibrinolytic activity, induction of chemotaxis and angiogenesis and inhibition of apoptosis (Yuan et al., 2015). Overexpression of *MDK* has been revealed in a variety of cancers, including gastric cancer (Xu et al., 2012), breast cancer (Ibusuki et al., 2009) and lung cancer (Hao et al., 2013). The Hippo pathway is an important pathway for organ growth, whose aberrant expression has been linked to tumorigenesis. The core kinases MST1/2 and LATS1/2 are tumour inhibitors that suppress the activity of the oncogenic factors Yes‐associated protein (YAP) and PDZ‐binding motif (TAZ) (Park, Shin, & Park, 2018), and their correlation with tumorigenesis, control of organ size and stem cell renewal has been reported (Park et al., 2018; Tao et al., 2017). This pathway has also been found in lung development and tumorigenesis (Yeung, Yu, & Yang, 2016). An article by Teoh & Das (2017) highlighted the role of the core members, upstream modulators and downstream effectors in lung cancer development and suggested that YAP and TAZ might be promising targets for future drug delivery and treatment.

In this study, we explored the functions of *miR‐188* in the biological characteristics of lung cancer stem cells with the involvement of *MDK* and the Hippo pathway.

## 2 METHODS

### 2.1 Ethical approval

All experimental procedures were performed in accordance with the guidelines by the Ethics Committee of the First Hospital of Jilin University (approval no. 2014‐243) and were confirmed to meet the principles and regulations described by Grundy (2015). Signed informed consent was obtained from all patients before the use of these clinical data for the study. The study conformed to the standards set by the *Declaration of Helsinki*, except for registration in a database. Animal studies were conducted in line with the principles and procedures approved by the Committee on the Ethics of Animal Experiments of the First Hospital of Jilin University (approval no. 2014‐007). Great efforts were made to minimize the number and pain of animals.

### 2.2 Clinical data collection

The First Hospital of Jilin University enrolled 75 patients with primary lung cancer from November 2014 to April 2015. The tumour tissues and adjacent normal tissues >5 cm away from the tumour tissues were resected during surgery. These patients, aged 59.68 ± 9.32 years (mean ± SD), included 43 men and 32 women. The clinical staging at the time of admission was as follows: 41 cases in stage I, 27 cases in stage II and seven cases in stage III. The size of the surgically removed tumour was 20.30 ± 11.59 mm. The resected tissue samples were cut into sections and embedded in paraffin, soaked in liquid nitrogen and preserved at −80°C. The patients were revisited every month to collect and record their prognosis. The duration of follow‐up visits was 4 years. None of these patients had received any preoperative chemotherapy or radiotherapy. The information on patients is given in Table 1.

**TABLE 1:** Demographic characteristics of the respondents

**Clinicopathological features**	***n* = 75**	
Age (years)	>60	*n* = 37
	≤60	*n* = 38
Sex	Male	*n* = 43
	Female	*n* = 32
Classification	I	*n* = 41
	II	*n* = 27
	III	*n* = 7

### 2.3 Cell culture and grouping

Lung cancer cell lines A549, H125, H520, CL1‐0 and H460, normal lung epithelial cell lines NuLi‐1 and BEAS‐2B and HEK293T cells were purchased from American Type Culture Collection (Manassas, VA, USA). Lung cancer cell lines were seeded in RPMI‐1640 medium (Gibco, Logan City, UT, USA) containing 10% fetal bovine serum; NuLi‐1 and HEK293T cells were cultured in DMEM medium (Gibco); and BEAS‐2B cells were cultured in LHC‐9 medium (Gibco). All cells were cultured in an incubator at 37°C in air enriched with 5% CO_2_.

### 2.4 Immunomagnetic bead separation

A549 and H125 cells were detached in 0.25% trypsin to make single‐cell suspension, centrifuged at 160 *g* for 2 min and resuspended in MACS Separation Buffer (Miltenyi Biotec, Auburn, CA, USA). Next, cells were mixed with 20 μl CD44 antibody magnetic bead marker (Miltenyi Biotec) and incubated at 4°C for 15 min. CD44^+^ cells were collected using an Auto MACS instrument (Miltenyi Biotec) and counted, then labelled with 100 μl CD133 antibody magnetic bead marker (Miltenyi Biotec) and incubated at 4°C for 45 min. The CD133^+^/CD44^+^ cells were collected using the Auto MACS instrument, and the purity of CD133^+^/CD44^+^ cells was detected using a flow cytometer (Attune NxT; Thermo Fisher Scientific Inc., Waltham, MA, USA).

### 2.5 Cell transfection

The *miR‐188* mimic, *miR‐188* control, *MDK* and negative control (NC) were purchased from Life Technologies (Grand Island, NY, USA). A549 and H125 cells were seeded into RPMI‐1640 medium and subjected to transfection when they reached a confluence of 70–90%. DNA was diluted with Opti‐MEM medium to prepare a DNA master mix. The diluted Lipofectamine 3000 reagent in each tube was supplemented with P3000 reagent at a ratio of 1:1. Subsequently, cells were mixed with DNA–lipid complexes and incubated for 3 days at 37°C. The lentiviral vector PGLV1 was purchased from GenePharma Co., Ltd (Shanghai, China). Cells were observed under a microscope (LIOOS600T; Shanghai Optical Instrument Factory, Shanghai, China) after transfection and collected for subsequent experiments. A Hippo‐specific inhibitor, XMU‐MP‐1, was purchased from MedChemExpress (Monmouth Junction, NJ, USA). A549 and H125 cells in the logarithmic growth phase were detached in 0.25% trypsin to prepare a single‐cell suspension. Next, 3 μm XMU‐MP‐1 was dissolved in 10 mm DMSO solution and added to the cell suspension to achieve a 1% concentration at 37°C for 24 h. Finally, the cells were washed in PBS and cultivated in RPMI‐1640 medium for further use (Fan et al., 2016).

### 2.6 Flow cytometry

The cultured cells (1 × 10^6^) were centrifuged, suspended in 0.3 ml PBS containing 10% calf serum and transferred into a 1.5 ml EP tube. The cells were fixed with 0.7 ml absolute ethanol at −20°C for >24 h. The cells were centrifuged at 500 *g* for 30 s and the supernatant was discarded. Thereafter, cells were resuspended with l ml PBS, centrifuged again, and the supernatant was discarded again. Precipitated cells were suspended in 100 μl of 1 mg/ml RNase A, incubated with 400 μl of 50 μg/ml annexin V–fluorescein isothiocyanate (FITC) for 15 min in the dark, followed by 400 μl of 50 μg/ml propidium iodide (PI) for 10 min devoid of light. Cell apoptosis was evaluated using a flow cytometer (Attune NxT; Thermo Fisher Scientific). Three independent experiments were performed.

### 2.7 Microarray analysis

The total miRNA molecules were randomly extracted from lung cancer tissues and adjacent tissues of three stage III patients using TRIzol Reagent (Invitrogen, Carlsbad, CA, USA). A miRNA Complete Labeling and Hyb Kit (Agilent Technologies, Santa Clara, CA, USA) was used for hybridization and labelling, human miRNA Microarray Release 14.0, 8 × 15K (Agilent Technologies) for purification and Agilent SureScan Dx (Agilent Technologies) for scanning. The analysis results were evaluated. Background correction and normalization of raw data were performed using robust multichip analysis. Student's paired *t* test was used to screen out miRNAs, *P* < 0.05, |log Fold change| > 2 was defined as differential miRNAs and plotted into a heatmap according to hierarchical clustering. Three independent experiments were performed.

### 2.8 Reverse transcription–quantitative polymerase chain reaction (RT‐qPCR)

TRIzol reagent (Invitrogen) was implemented to extract total RNA from lung cancer tumour tissues and adjacent tissues. A PrimeScript Reagent Kit (TAKARA, Otsu, Shiga, Japan) was used for reverse transcription and to amplify complementary DNA. The qPCR was conducted with QuantStudio 3 and 5 Real‐Time PCR Systems (ThermoFisher Scientific). Gene expression was quantified using a TaqMan Fast Advanced Master Mix Kit (ThermoFisher Scientific). All samples were subjected to three independent analyses. The analysis results were based on the −2^ΔΔ^ *^Ct^* method. The relevant primers are shown in Table 2. Three independent experiments were performed.

**TABLE 2:** The primer sequence for RT‐qPCR

**Gene**	**Primer sequence**
*U6*	F: 5′‐CAAATTCGTGAAGCGTTCCATA‐3′
	R: 5′‐GTGCAGGGTCCGAGGTATTC‐3′
*GAPDH*	F: 5′‐AAGGTCGGAGTCAACGGATTT‐3′
	R: 5′‐CCTGGAAGATGGTGATGGGAT‐3′
*miR‐188*	F: 5′‐CATCCCTTGCATGGTGGAGGG‐3′
	R: 5′‐GTGCAGGGTCCGAGGTATTC‐3′
*MDK*	F: 5′‐CTGGGGGCTGGATTCGGGGGTGGG‐3′
	R: 5′‐ACCCCCACCCCCGAATCCAGCCCCCA‐3′
*YAP*	F: 5′‐GGUGAUACUAUCAACCAAATT‐3′
	R: 5′‐GGAUACAGGAGAAAACGCATT‐3′

Abbreviations: F, forward; *GAPDH*, glyceraldehyde‐3‐phosphate dehydrogenase; *miR‐188*, microRNA‐188; *MDK*, midkine; R, reverse; RT‐qPCR, reverse transcription–quantitative polymerase chain reaction; U6, small nuclear RNA; *YAP*, Yes‐associated protein.

### 2.9 Cell counting kit‐8 assay

The cultured cells were detached with a mixture of 0.02% EDTA and 0.25% trypsin to prepare A549 and H125 cell suspensions. The cell suspension (100 μl) was added to each well in a 96‐well plate and cultured in an incubator for 24 h. Next, 10 μl of cell counting kit‐8 (CCK‐8) reagent (TAKARA) was added to each well, and the cells were cultured for 2 h. The optical density (OD) value of each well was measured at 460 nm. Three independent experiments were performed.

### 2.10 Transwell assays

In aseptic conditions, the apical chambers were precoated with Matrigel (BD Biosciences, Franklin Lakes, NJ, USA) for 30 min. Next, each chamber was loaded with 30 μl serum‐free RPMI‐1640 medium and placed in an incubator in air enriched with 5% CO_2_ for later use. The cells were trypsinized, centrifuged, resuspended in serum‐free medium and diluted to a cell suspension of 5 × 10^5^ cells ml^−1^. Next, the basolateral chamber of the Transwell was supplemented with 500 μl RPMI‐1640 medium containing 10% fetal bovine serum, and then 200 μl cell suspension was added to each apical chambe. The Transwell plates were incubated at 37°C in air enriched with 5% CO_2_ for 48 h. After that, the cells in the inner membrane were washed using PBS. The remaining invaded cells were stained with Crystal Violet solution for 10 min, and photographs of the cells were captured under the microscope. Cell migration was measured in a similar manner without precoating with Matrigel in the apical chambers.

### 2.11 Xenograft transplantation model

Forty‐eight female specific pathogen‐free mice (4–6 weeks of age, weighing 20 ± 2 g) were purchased from Beijing Vital River Laboratory Animal Technology Co., Ltd. (Beijing, China) and randomly divided into 16 groups [(*miR‐188* mimic and *miR‐188* control, *MDK* and NC, *miR‐188* mimic + *MDK* and *miR‐188* mimic + NC, XMU‐MP‐1 and DMSO) × (A549/H125)], with three mice in each group. A total of 4 × 10^6^ cells in each group were dispersed in 2 ml physiological saline and injected s.c. into each group of nude mice. The tumour volume in the mice was measured every 7 days after injection. The tumour volume = *L* × *W* ^2^/2, where *L* indicates the length and *W* the width of tumours (Tang et al., 2019). After 28 days, mice were killed by overdose of pentobarbital sodium (120 mg kg^−1^, i.p.), and the tumours were excised and weighed.

### 2.12 Luciferase activity assay

The 3′ UTR binding sequence of *miR‐188* and *MDK* was predicted with the online prediction software StarBase (http://starbase.sysu.EdU.cn/). The wild‐type (WT) and mutant‐type (MUT) sequences of the *MDK* 3′ UTR binding sequence were synthesized by Sangon Biotech Co., Ltd (Shanghai, China) and inserted into pMIR‐REPORT luciferase reporter vector (Thermo Fisher Scientific) (Jiang et al., 2014). A Lipofectamine 3000 transfection kit (Invitrogen) was used to co‐transfect WT plasmid, MUT plasmid with *miR‐188* or mimic NC into cells. Cells were lysed after 24 h, and the intensity of luciferase activity was measured using a dual‐luciferase reporter assay system (Promega Corporation, Madison, WI, USA; intensity = RLU1/RLU2, where RLU1 is the firefly luciferase reaction intensity and RLU2 the renilla luciferase reaction intensity) (Jiang et al., 2014).

### 2.13 Western blot analysis

The tumour tissue homogenate was treated with 1 ml of lysate (RIPA Lysis Buffer; Applygen Technology Co., Ltd, Beijing, China) and the lysate was transferred to a 1.5‐ml centrifuge tube, followed by centrifugation at 2000 *g* at 4°C for 5 min. The supernatant was taken for SDS‐PAGE separation, and the separated gel was electroblotted on polyvinylidene fluoride membranes (EMD Millipore Corp., Billerica, MA, USA). After that, the membranes were blocked with dried skimmed milk and probed with the corresponding primary antibodies against YAP (1:10,000; ab181602), TAZ (1:500; ab84927) and GAPDH (1:10,000, ab181602; all from Abcam, Cambridge, UK) and then with the secondary antibody (1:3000; ab205718; Abcam). ImageJ software (v.1.8.0; US National Institutes of Health, Bethesda, MD, USA) was used to perform quantitative optical density analysis on the immunoblot images.

### 2.14 Statistical analysis

Statistical analysis was carried out with SPSS v.22.0 software (SPSS Inc., Chicago, IL, USA). Measurement data were depicted as the mean ± SD. Student's paired *t* test was used for comparisons between two groups. One‐ or two‐way ANOVA was adopted for comparisons among multiple groups, after which Tukey's multiple comparisons test was applied for pairwise comparisons. The survival curve was calculated using the Kalpan–Meier method, and the post‐statistical analysis was performed using the log rank test. A value of *P* < 0.05 was regarded as statistical significance.

## 3 RESULTS

### 3.1 MicroRNA‐188, beneficial to the patient's prognosis, is downregulated in lung cancer tissues and cells

**FIGURE 1:**
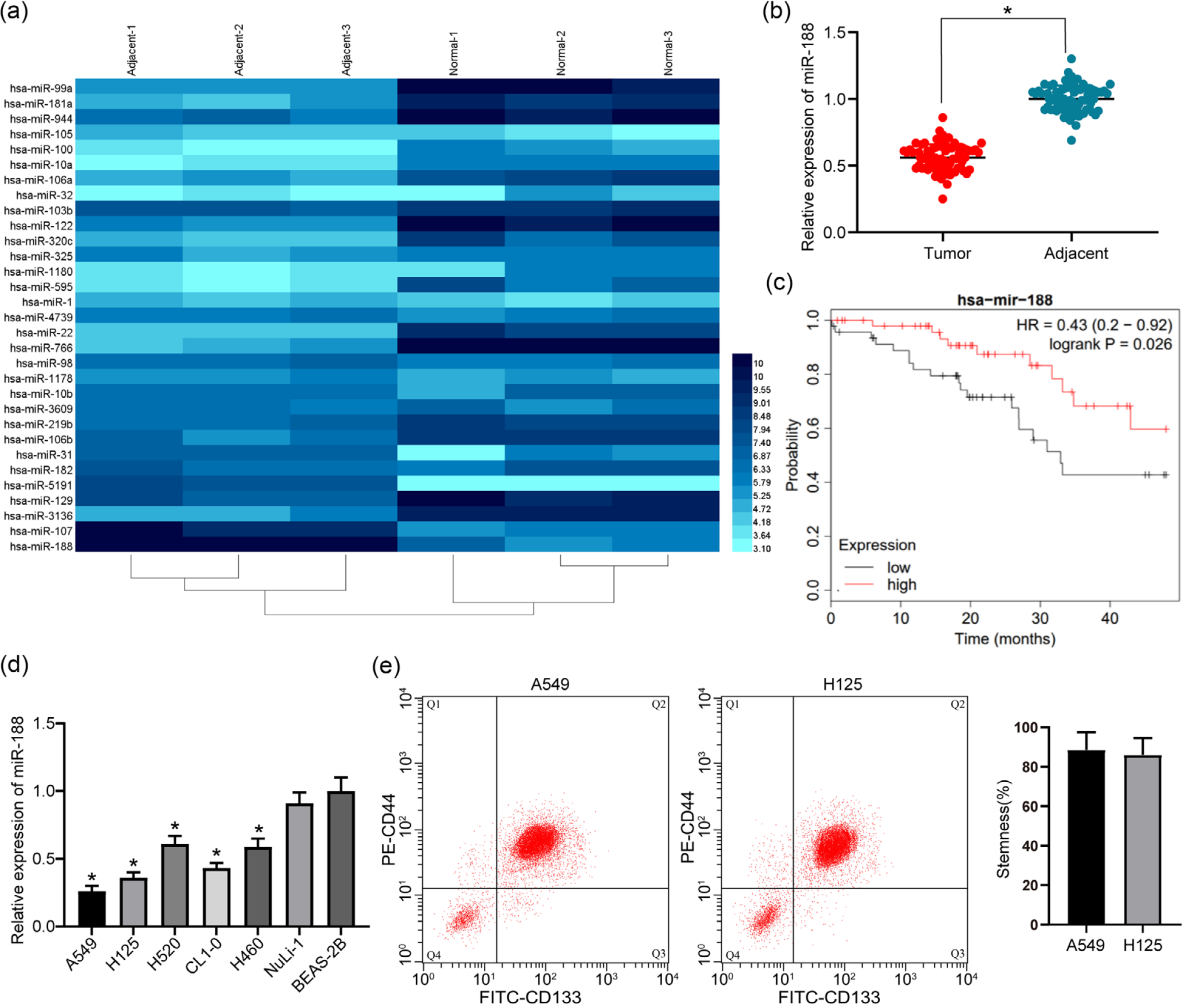
MicroRNA‐188 (*miR‐188*) is downregulated in lung cancer tissues and cells. (a) MicroRNA microarray analysis of tumour tissues and adjacent tissues in three lung cancer patients. (b) RT‐qPCR detection of *miR‐188* expression in tumour tissues and adjacent tissues of 75 patients with lung cancer (^*^ *P* < 0.05 according to two‐way ANOVA). (c) Correlation analysis between *miR‐188* expression and survival rate of lung cancer patients. (d) RT‐qPCR detection of *miR‐188* expression in lung cancer tumour cell lines and normal lung epithelial cell lines (^*^ *P* < 0.05 according to two‐way ANOVA). (e) Flow cytometry detection of the stemness of tumour stem cells isolated from A549 and H125 cells (with CD44 and CD133 as markers)

MicroRNA‐188 was found to be reduced in lung cancer tissues by examining miRNA microarray differences between tumour tissues and adjacent normal tissues in lung cancer patients (Figure 1a). This result was validated using the RT‐qPCR (Figure 1b). The survival of lung cancer patients was analysed and showed that *miR‐188* expression was positively correlated with the survival rate of lung cancer patients (Figure 1c). We also found that *miR‐188* was poorly expressed in lung cancer tumour cells relative to the BEAS‐2B cells (Figure 1d). Next, the stem cells from A549 and H125 cell clusters were isolated by immunomagnetic beads for subsequent experiments (Figure 1e).

### 3.2 Overexpression of *miR‐188* inhibits lung cancer stem cell activity

**FIGURE 2:**
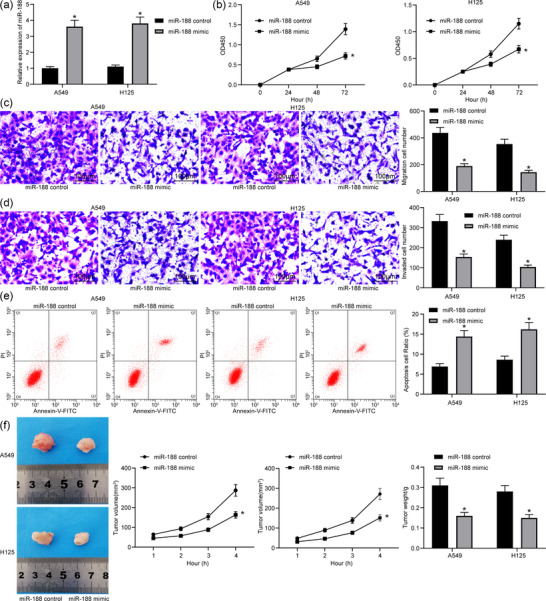
Overexpression of *miR‐188* inhibits lung cancer stem cell activity. (a) Detection of *miR‐188* expression in cells with *miR‐188* mimic by RT‐qPCR (^*^ *P* < 0.05 according to two‐way ANOVA). (b) Cell counting kit‐8 assay was used to detect change in cell proliferation in cells with *miR‐188* mimic (^*^ *P* < 0.05 according to two‐way ANOVA). (c) Transwell assay was used to detect change in cell migration in cells with *miR‐188* mimic (^*^ *P* < 0.05 according to two‐way ANOVA). (d) Transwell assay was used to detect change in cell invasion in cells with *miR‐188* mimic (^*^ *P* < 0.05 according to two‐way ANOVA). (e) Flow cytometry was used to detect change in cell apoptosis in cells with *miR‐188* mimic [with propidium iodide (PI) and annexin V as markers; ^*^ *P* < 0.05 according to two‐way ANOVA]. (f) Changes in lung tumour volume and weight in mice in xenoplastic transplantation of *miR‐188* mimic (^*^ *P* < 0.05 according to two‐way ANOVA)

The cancer stem cells were collected and treated with *miR‐188* mimic, and the transfection was successfully performed according to the RT‐qPCR results (Figure 2a). The CCK‐8 results demonstrated that the OD value at 460 nm was reduced after *miR‐188* overexpression (Figure 2b). In addition, the number of migrated cells (Figure 2c) and the number of invading cells (Figure 2d) at 48 h was reduced according to the Transwell assays. The flow cytometry results showed that the number of annexin V–FITC/PI‐positive cells was increased (Figure 2e). In addition, xenoplastic transplantation of stem cells overexpressing *miR‐188* also presented retarded tumour growth rate and reduced tumour weight at 28 days (Figure 2f).

### 3.3 MicroRNA‐188 targets *MDK*

**FIGURE 3:**
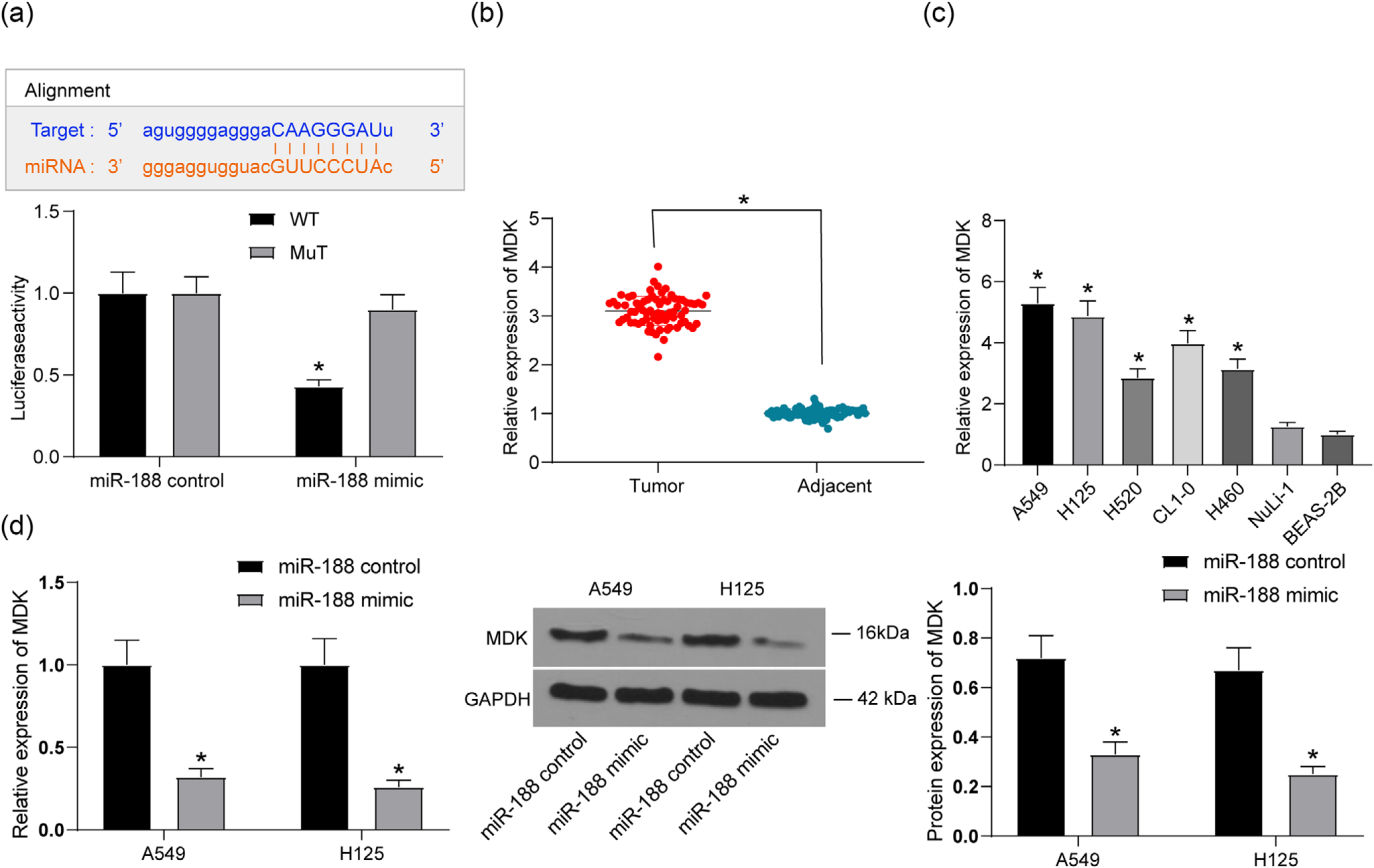
MicroRNA‐188 (*miR‐188*) targets *MDK*. (a) StarBase predicted the binding sites of *miR‐188* and *MDK*, and the luciferase activity test validated this result (*P < 0.05 according to two‐way ANOVA). (b) Detection of *MDK* expression in lung cancer tissues by RT‐qPCR (^*^ *P* < 0.05 according to two‐way ANOVA). (c) Detection of *MDK* expression in lung cancer cell lines and normal lung epithelial cell lines by RT‐qPCR (^*^ *P* < 0.05 according to one‐way ANOVA). (d) *MDK* expression in cancer stem cells after *miR‐188* overexpression determined by RT‐qPCR and western blot analysis (^*^ *P* < 0.05 according to two‐way ANOVA)

We predicted *MDK* as a target mRNA of *miR‐188* on StarBase, and this binding relationship was validated using a dual luciferase assay. The results suggested that co‐transfection of pMIR‐MDK‐WT vector and *miR‐188* mimic led to a significant decline in luciferase activity in cells, whereas other co‐transfection showed no major changes (Figure 3a), and *MDK* expression was increased in lung cancer tissues (Figure 3b) and was also elevated in the lung cancer cell lines (Figure 3c). In addition, expression of *MDK* in cancer stem cells was determined after *miR‐188* mimic administration. We found that the mRNA and protein expressions of *MDK* were notably increased after *miR‐188* overexpression (Figure 3d), indicating that *MDK* is a downstream transcript of *miR‐188* and fulfils key functions in lung cancer progression.

### 3.4 Overexpression of *MDK* induces lung cancer stem cell activity

**FIGURE 4:**
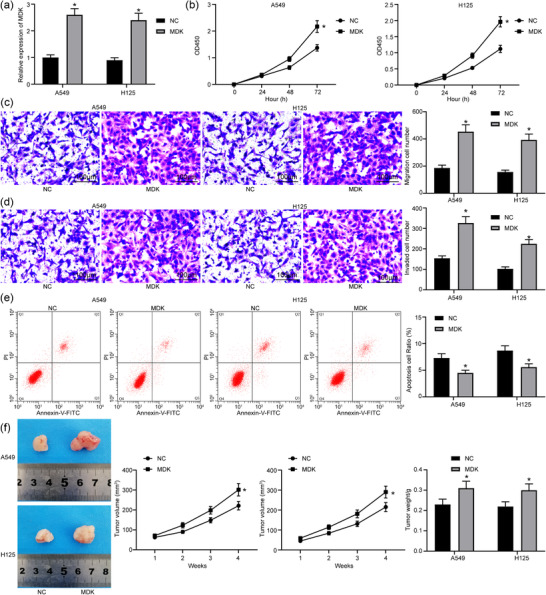
Overexpression of *MDK* promotes lung cancer stem cell activity. (a) Detection of *MDK* expression in cells with *MDK* by RT‐qPCR (^*^ *P* < 0.05 according to two‐way ANOVA). (b) Cell counting kit‐8 assay was used to detect change in cell proliferation in cells with *MDK* (^*^ *P* < 0.05 according to two‐way ANOVA). (c) Transwell assay was used to detect change in cell migration in cells with *MDK* (^*^ *P* < 0.05 according to two‐way ANOVA). (d) Transwell assay was used to detect change in cell invasion in cells with *MDK* (^*^ *P* < 0.05 according to two‐way ANOVA). (e) Flow cytometry was used to detect change in cell apoptosis in cells with *MDK* [with propidium iodide (PI) and annexin V as markers; ^*^ *P* < 0.05 according to two‐way ANOVA]. (f) Changes in lung tumour volume and weight in mice in xenoplastic transplantation of *MDK* (^*^ *P* < 0.05 according to two‐way ANOVA)

Overexpression of *MDK* was introduced in cancer stem cells through lentiviral vectors (Figure 4a). It was found that the viability of cancer stem cells was enhanced (Figure 4b), the number of migrated cells (Figure 4c) and invading cells (Figure 4d) was increased, and the number annexin V–FITC/PI‐positive cells was reduced (Figure 4e). In addition, xenoplastic transplantation of stem cells overexpressing *MDK* elevated the tumour growth rate and the weight of the tumour at 28 days (Figure 4f).

### 3.5 Overexpressed *MDK* partly inhibits the biological effects of overexpressed *miR‐188*

**FIGURE 5:**
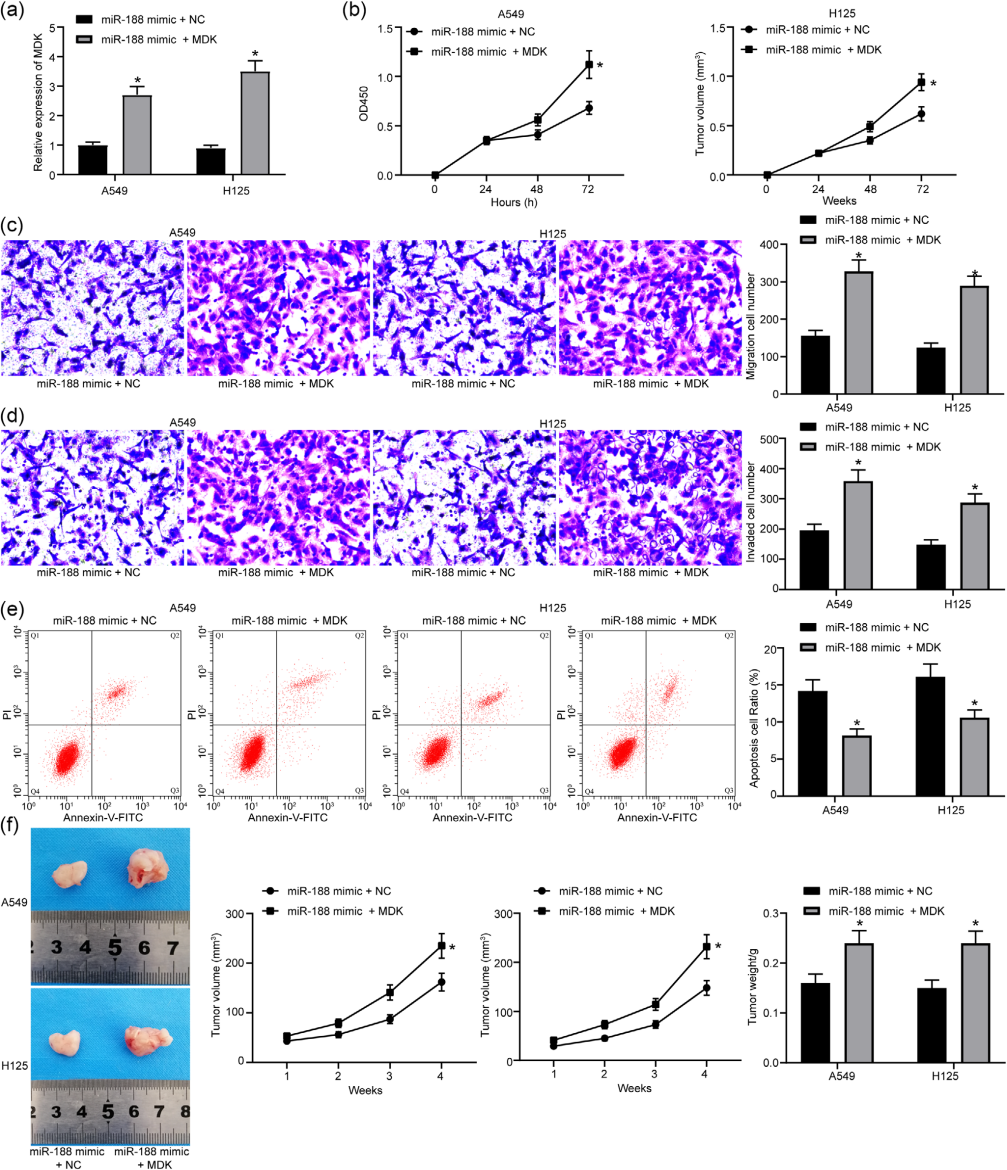
Overexpression of *MDK* partly inhibits the biological effects of overexpressed *miR‐188*. (a) Detection of *MDK* expression in cells with *miR‐188* mimic + *MDK* by RT‐qPCR (^*^ *P* < 0.05 according to two‐way ANOVA). (b) Cell counting kit‐8 assay was used to detect change in cell proliferation in cells with *miR‐188* mimic + *MDK* (^*^ *P* < 0.05 according to two‐way ANOVA). (c) Transwell assay was used to detect change in cell migration in cells with *miR‐188* mimic + *MDK* (^*^ *P* < 0.05 according to two‐way ANOVA). (d) Transwell assay was used to detect change in cell invasion in cells with *miR‐188* mimic + *MDK* (^*^ *P* < 0.05 according to two‐way ANOVA). (e) Flow cytometry was used to detect change in cell apoptosis in cells with *miR‐188* mimic + *MDK* [with propidium iodide (PI) and annexin V as markers; ^*^ *P* < 0.05 according to two‐way ANOVA]. (f) Changes in lung tumour volume and weight in mice in xenoplastic transplantation of *miR‐188* mimic + *MDK* (^*^ *P* < 0.05 according to two‐way ANOVA)

To validate the involvement of *MDK* in *miR‐188*‐mediated events, a rescue experiment was performed, in which cells transfected with *miR‐188* mimic were also transfected with lentiviral vectors overexpressing *MDK*, and the efficiency of transfection was validated by RT‐qPCR (Figure 5a). Compared with administration of *miR‐188* mimic alone, we found that further upregulation of *miR‐188* enhanced cell viability (Figure 5b), increased the number of migrated cells (Figure 5c) and invading cells (Figure 5d) and decreased the number of annexin V–FITC/PI‐positive cells (Figure 5e). In addition, xenoplastic transplantation of cells co‐transfected with *miR‐188* mimic and *MDK* vector led to an increase in the tumour growth rate and the the weight of the tumour at 28 days compared with xenoplastic transplantation of cells co‐transfected with *miR‐188* mimic alone (Figure 5f).

### 3.6 MicroRNA‐188 mediates Hippo pathway activity

**FIGURE 6:**
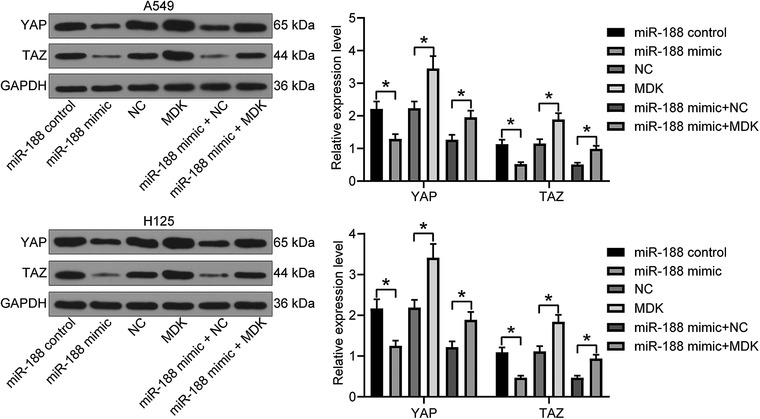
MicroRNA‐188 (*miR‐188*) mediates Hippo pathway activity. The protein levels in the Hippo pathway in cells with *miR‐188* mimic and *miR‐188* control, *MDK* and negative control (NC), *miR‐188* mimic + NC and *miR‐188* mimic + *MDK* were determined by western blot analysis

The protein levels of key kinases of the Hippo pathway, YAP and TAZ, in cells were determined using western blot analysis. It was found that the levels of activated YAP and TAZ were increased in lung cancer tissues. Overexpression of *miR‐188* was found to activate this signalling by suppressing the levels of YAP and TAZ, whereas the levels of these kinases were recovered by the further upregulation of *MDK* (Figure 6).

### 3.7 Suppression of Hippo pathway promotes lung cancer stem cell activity

**FIGURE 7:**
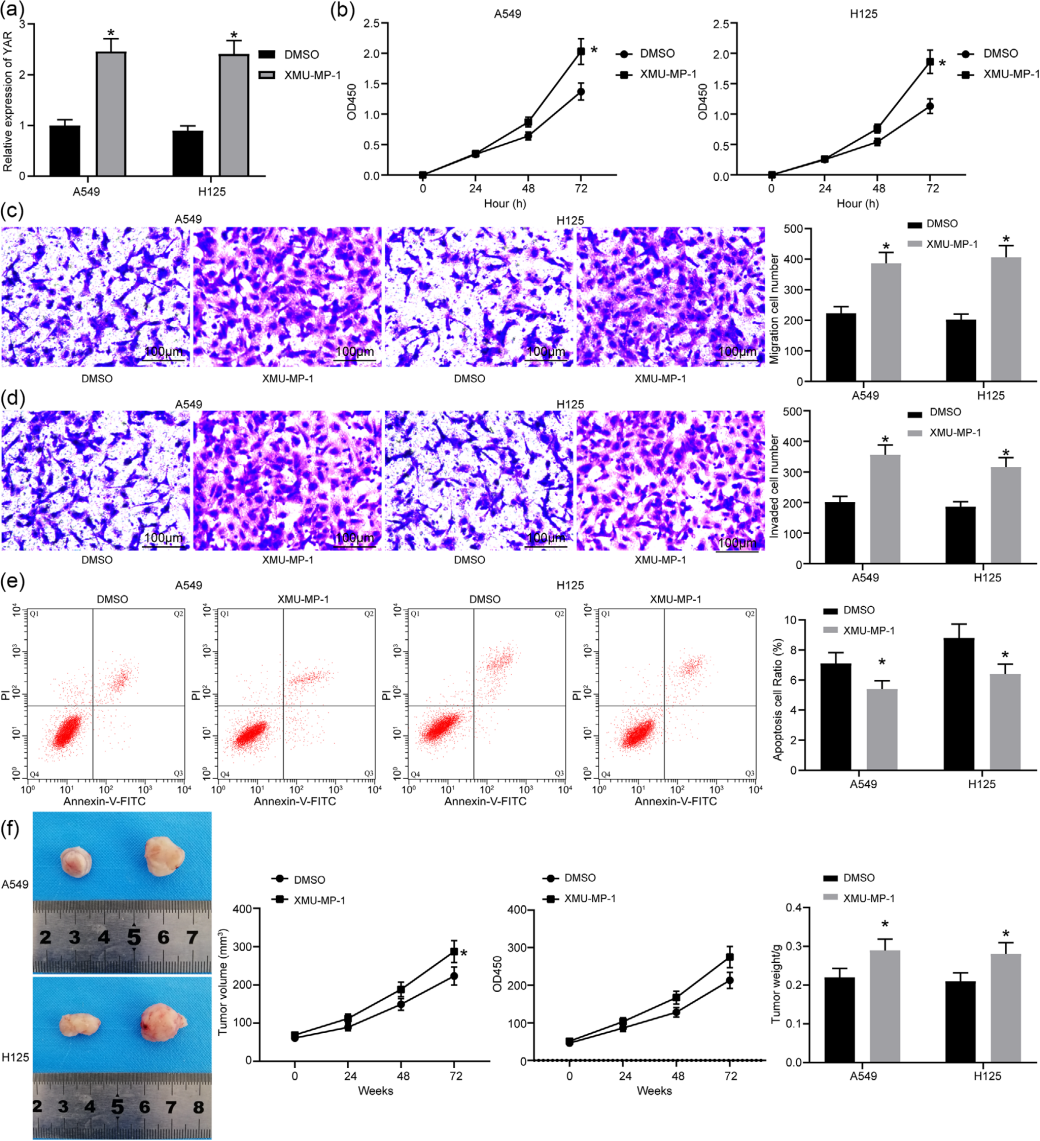
Suppression of Hippo pathway induces lung cancer stem cell activity. (a) Detection of YAP expression in cells transfected with XMU‐MP‐1 by RT‐qPCR (^*^ *P* < 0.05 according to two‐way ANOVA). (b) Cell counting kit‐8 assay was used to detect change in cell proliferation in cells transfected with XMU‐MP‐1 (^*^ *P* < 0.05 according to the two‐way ANOVA). (c) Transwell assay was used to detect change in cell migration in cells transfected with XMU‐MP‐1 (^*^ *P* < 0.05 according to two‐way ANOVA). (d) Transwell assay was used to detect change in cell invasion in cells transfected with XMU‐MP‐1 (^*^ *P* < 0.05 according to two‐way ANOVA). (e) Flow cytometry was used to detect change in cell apoptosis in cells transfected with XMU‐MP‐1 [with propidium iodide (PI) and annexin V as markers; ^*^ *P* < 0.05 according to two‐way ANOVA]. (f) Changes in lung tumour volume and weight in mice after xenoplastic transplantation of cells transfected with XMU‐MP‐1 (^*^ *P* < 0.05 according to two‐way ANOVA)

To validate the roles of the Hippo signalling pathway in cancer stem cell growth and tumorigenesis of lung cancer, a Hippo‐specific inhibitor, XMU‐MP‐1, was administered into the stem cells (Figure 7a). Consequently, it was found that the malignant behaviours of the cells were promoted, presenting as enhanced cell viability (Figure 7b), increased cell migration (Figure 7c) and cell invasion (Figure 7d) and a reduction in the apoptosis rate in cells (Figure 7e). Implantation of cells treated with XMU‐MP‐1 in nude mice led to an increase in tumour growth *in vivo* (Figure 7f). These results validated the suppressing roles of the Hippo pathway in stem cell viability and tumour growth of lung cancer.

## 4 DISCUSSION

Understanding new molecular mechanisms is of great importance in helping to develop potential therapeutic strategies for cancer. Here, we performed this research to elucidate the biological effects of *miR‐188* on lung cancer and its specific molecular mechanisms. Collectively, the present study demonstrated that *miR‐188* played a suppressive role in the biological characteristics of lung cancer stem cells via targeting the *MDK*‐mediated Hippo pathway.

Initially, we found a poor expression profile of *miR‐188* in tumour tissues and in the separated cancer stem cells. Next, *miR‐188* mimic was introduced into cells, after which overexpression weakened lung cancer stem cell proliferation, invasion and tumour formation rate and enhanced apoptosis. The suppressive function of *miR‐188* in human diseases has been discussed before. For instance, *in vitro* transfection of *miR‐188* restricted NSCLC cell proliferation potential and stimulated cell apoptosis, in addition to suppressing tumour growth in a xenograft model (Zhao et al., 2018). Another study revealed that *miR‐188* was downregulated in lung adenocarcinoma and that upregulation of *miR‐188* inhibited proliferation and resistance to apoptosis of lung adenocarcinoma cells (Lv et al., 2020). Furthermore, *miR‐188*‐5p enhanced the growth of gastric cancer cells *in vitro* and tumour metastasis *in vivo* (Li et al., 2019).

In addition, StarBase prediction and luciferase activity analysis suggested that *miR‐188* targeted *MDK*. Many miRNA targets have been characterized in different types of cancers. *miR‐188* was reduced in prostate cancer and retarded cancer cell growth via repression of LAPTM4B (Zhang et al., 2015). Additionally, downregulation *miR‐188* was found in hepatocellular carcinoma, where it acted as a tumour suppressor through binding to

*FGF5* (Fang et al., 2015). It was also demonstrated that *miR‐188* inhibited oral squamous cell carcinoma by targeting *SIX1*, offering new insights into the detailed molecular mechanisms of oral squamous cell carcinoma (Wang & Liu, 2016). In the present study, we detected *MDK* expression in lung cancer tissues and cells, suggesting that *MDK* expression was increased in lung cancer, and overexpression of *MDK* promoted lung cancer stem cell activity. *MDK* is able to activate some signalling pathways and results in various cellular process through interaction with its downstream proteins (Muramatsu, 2011; Weckbach, Muramatsu, & Walzog, 2011). *MDK* has also been found to be important in the process of human carcinogenesis, which might be a potential target for cancer therapy. *MDK* downregulation suppressed glioma cell proliferation and the tumour growth in nude mice (Luo et al., 2015). Furthermore, upregulation of *MDK* might lead to the stemness properties and cell survival of prostate cancer stem cells (Erdogan, Doganlar, Doganlar, Turkekul, & Serttas, 2017).

Next, the protein levels of YAP and TAZ in lung cancer cells were determined, and the findings suggested that Hippo pathway activity was suppressed in lung cancer tissues. Additionally, we also found that suppression of the Hippo pathway promoted lung cancer stem cell activity. As already mentioned, YAP and TAZ are crucial oncogenes that are activated once Hippo is suppressed. On phosphorylation by LATS1/2, YAP is prevented from nuclear accumulation and consequently degraded in the cytoplasm, whereas non‐phosphorylated YAP accumulates in the nucleus and promotes aberrant cell proliferation (Wennmann et al., 2014). Recent advances in understanding the components and functional implications of the Hippo pathway indicate its roles in tumorigenesis, tissue regeneration, organ development and stem cell self‐renewal (Park et al., 2018). The Hippo signalling pathway has also been implicated in playing pivotal roles in lung cancer pathogenesis, including multidrug resistance and tumour development (Liu, Zuo, & Ou, 2018). Taking *in vivo* xenograft and *in vitro* cell culture approaches, Liu et al. (2016) showed that PDE/cGMP/PKG signalling maintains prostate cancer stem cell stemness by targeting to Hippo/TAZ pathway. Moreover, Bora‐Singhal et al. (2015) showed that YAP was enhanced in NSCLC stem‐like cells and resulted in their self‐renewal and ability to form angiogenic tubules.

**FIGURE 8:**
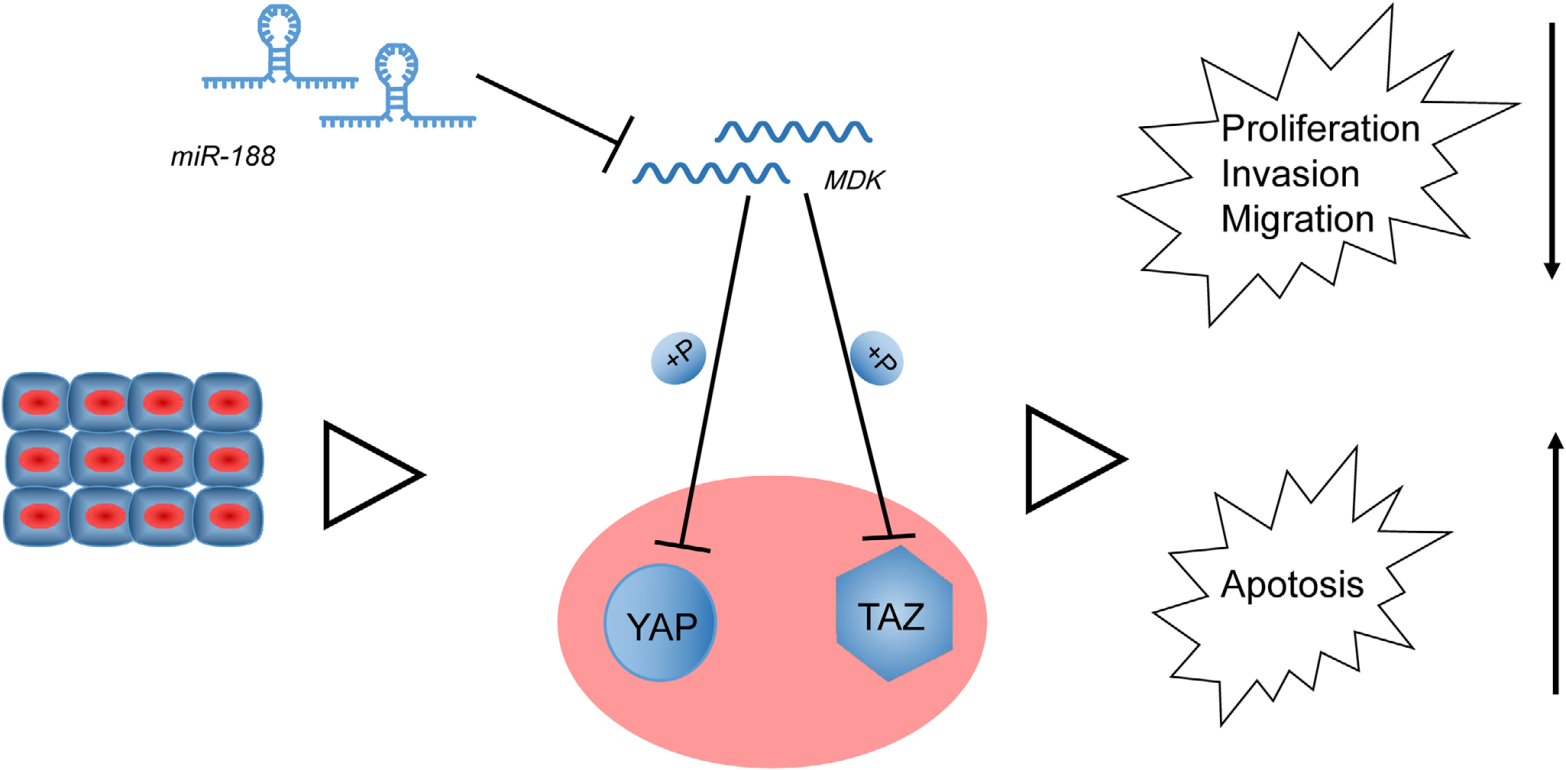
The mechanistic diagram depicts that *miR‐188* targets the *MDK*‐mediated Hippo pathway, inhibits the activation of YAP protein, weakens the proliferation, migration and invasion of lung cancer stem cells and enhances their apoptotic ability

In summary, our study highlights a new molecular mechanism involving *miR‐188*, *MDK* and the Hippo signalling pathway, which plays a suppressive role in biological activity of lung cancer stem cells (Figure 8). Exploration of the function of *miR‐188* not only enhances our understanding of lung cancer carcinogenesis but also identifies *miR‐188* as a promising biomarker for lung cancer diagnosis and therapy.

## COMPETING INTERESTS

None declared.

## AUTHOR CONTRIBUTIONS

Study design: X.Y. Experimental work: X.Y., B.W. and W.C. Data analysis: X.Y., B.W. and W.C. Writing the manuscript: X.Y. Overall coordination and research governance: X.M. All authors read and approved the final version of the manuscript and agree to be accountable for all aspects of the work in ensuring that questions related to the accuracy or integrity of any part of the work are appropriately investigated and resolved. All persons designated as authors qualify for authorship, and all those who qualify for authorship are listed.

## Data Availability

The data that support the findings of this study are available from the corresponding author upon reasonable request.
